# Investigation on Preparation and Properties of Crack Sealants Based on CNTs/SBS Composite-Modified Asphalt

**DOI:** 10.3390/ma14164569

**Published:** 2021-08-14

**Authors:** Yafeng Gong, Yunze Pang, Feng He, Haipeng Bi

**Affiliations:** College of Transportation, Jilin University, Changchun 130025, China; gongyf@jlu.edu.cn (Y.G.); pangyz19@mails.jlu.edu.cn (Y.P.); bihp@jlu.edu.cn (H.B.)

**Keywords:** pavement engineering, modified asphalt sealants, CNTs, LAS test, fatigue resistance performance

## Abstract

Crack is the main distress of asphalt pavement. Sealant is one of the most commonly used crack repair materials, and its performance is the key to affect the service life of asphalt pavements. In order to find an efficient modifier and optimize the performances of crack sealants. In this paper, carbon nanotubes (CNTs) and styrene-butadiene-styrene (SBS) were used as modifiers to prepare CNTs/SBS composite-modified asphalt crack sealant. The properties of the sealant were tested to evaluate its suitability for crack repair, which included the viscosity, softening point, resilience recovery, cone penetration, flow value, penetration, aging resistance, and fatigue resistance. The results showed that the conventional properties of the sealants meet the requirements of the specification. In addition, after heating aging, the elastic recovery rate of the sealant containing more CNTs decreased only slightly. The sealant containing 1 wt% CNTs exhibited a higher viscosity, fatigue resistance, thermal aging resistance.

## 1. Introduction

Crack is one of the most common distress in asphalt pavement, which is gradually aggravated under the influence of natural environment and vehicle load, eventually leading to damaged road surface [1]. Among many asphalt pavement crack repair materials, hot-poured sealant has been widely used because of its high cost effectiveness and good road performance [2]. However, the problems such as aging, fatigue, and lack of thermal stability of asphalt-based sealants will cause sealants to fail or even crack again [3]. Therefore, it is very necessary to prepare a kind of asphalt pavement crack sealant with excellent performance.

In order to prepare sealant with excellent performance, adding modifiers to sealant is a common method to improve sealant performance. Zhang et al. [3] developed a new type of concrete pavement sealant, where styrene-butadiene-styrene (SBS) was selected as the modifier. It is found that SBS can form a stable network structure in asphalt and improve the flexibility of asphalt. However, the performance of single polymer-modified asphalt was still not perfect [4,5]. Although SBS polymer can improve the stiffness of the asphalt binder, the butadiene structure in its molecular chain lacks thermal aging resistance [6]. In recent years, much attention has been paid to the research on the use of nanotechnology to improve the performance of pavement building materials [7]. Some researchers have proposed that nanomaterials such as carbon nanotubes (CNTs), nano-clay, and nano-titanium dioxide was added to asphalt as modifiers to change the macroscopic properties of materials on a nanometer scale [8]. Among them, CNTs has the advantages of a lightweight, good stability, and high strength, so it has shown great potential to reduce the aging degree and improve the thermal stability in the field of modified asphalt materials [9]. Wang et al. [10] and Amin et al. [11] studied the aging and rheological properties of multi-walled CNTs-modified asphalt. It was found that multi-walled CNTs had excellent thermal-oxidative aging resistance and UV oxidative aging resistance, and enhanced the high temperature damage resistance of modified asphalt. Yang et al. [12] used scanning electron microscope (SEM) to observe the micro-morphology of CNTs and asphalt. It was found that the intertwined CNTs forms a network, and parts of the CNTs are connected to the asphalt, which can delay the destruction of the asphalt interface. Considering that the nanomaterials tend to agglomerate, the content of CNTs is also very important. Staub de Melo et al. [13] found that when the content of CNTs reached 3%, the rheological properties of asphalt binders decreased due to the agglomeration of the modifiers. In terms of fatigue resistance performance, Santagata et al. [14] believed that fully dispersed CNTs can significantly enhance the fatigue resistance of asphalt materials, but the effectiveness of modifiers was highly dependent on the physical and chemical properties of base asphalt [15]. In addition, Santagata et al. [16] also found that the anti-fatigue effectiveness of nano-modifiers highly depends on the properties of nano-materials and test conditions.

In addition, the addition of CNTs can produce a synergistic effect with the polymer, which plays a positive role in improving the performance of the modified asphalt [17]. Wang et al. [18] modified asphalt binders with CNTs/SBS composite material, and the test results showed that CNTs can improve the aging resistance and high-temperature rheological properties of asphalt binders. Wang et al. [19] through the methods of fluorescence microscope, scanning electron microscope, and molecular dynamics simulation, found that CNTs can transport small molecules in asphalt and improve the swelling degree of the polymer. Wang et al. [20] found that CNTs can adsorb saturates, aromatics, and resins in SBS-modified asphalt by molecular dynamics simulation, thus strengthening the interface between polymer and asphalt. In terms of engineering cost, although the initial cost of adding CNTs as a modifier to pavement materials is relatively high, with the use and consumption of pavements, it can greatly reduce the cost of pavements maintenance and prolong the service life of pavements [21,22]. Moreover, the preparation cost of CNTs will continue to reduce with the development of engineering technology, so it has a great development prospect.

Previous studies have shown that CNTs have strong applicability in asphalt materials, and can make up for the deficiency of a single modification of SBS. However, a series of studies on the properties of the developed sealant is needed to evaluate its applicability to crack repair. At present, the research on the properties of sealants is mainly focused on the anti-aging properties and bonding properties of sealants. Cao et al. [2] studied the aging behavior of the finished sealant and found that during the aging process, the polymer in the sealant was degraded, which hardened the sealant and finally formed cracks. Li et al. [23], in order to systematically evaluate the field performance of sealants, selected nine kinds of sealants commonly used in northern China for the heating aging test, and the mass loss, taper penetration ratio, and softening point after aging were used as evaluation indexes. Sun et al. [24] based on the surface energy theory, studied how to quantitatively evaluate the adhesion between sealants and crack walls formed by different types of asphalt mixtures. Due to the influence of traffic load, fatigue cracking of asphalt concrete pavement is a major disease, and the corresponding sealant also appears more fatigue damaged. However, there is a lack of research on the fatigue performance of sealants, so this paper draws lessons from the research method of fatigue characteristics of asphalt as the basis and reference for the study of fatigue performance of sealants [25].

There are many methods to study the fatigue properties of asphalt materials. Strategic Highway Research Program (SHRP) proposes to use the product of complex shear modulus and phase angle sine |G*|∙sinδ as a parameter to evaluate the fatigue performance of asphalt binders. This parameter is measured in the linear viscoelastic region of the material, and cannot have a good correlation with the fatigue damage of modified asphalt. However, a suitable fatigue damage criterion should be applicable to a wide range of loading environment, so this evaluation standard has been questioned by many researchers [26,27,28]. At present, time scan test (TS) and linear amplitude scan test (LAS) are two widely accepted methods. Bessa et al. [29] and Notani et al. [30] have studied the two methods systematically. The TS test is loaded at a constant frequency and temperature in a controlled stress or strain mode. However, the time period of the TS test is difficult to predict, and the test results are highly dependent on the loading amplitude, so this experimental method has some limitations [31]. LAS test is an accelerated fatigue test method. The LAS test data are analyzed by simplified viscoelastic continuous damage (S-VECD) model to predict the fatigue damage evolution of asphalt [32]. The content of LAS test is divided into two parts. The first part is to obtain the undamaged rheological parameters of asphalt binders by frequency sweep test, and the second part is to obtain the fatigue properties of asphalt binders by linearly increased amplitude loading. This method can predict the fatigue life under any loading condition and has the advantage of simple operation and short time consumption.

Therefore, in view of the positive role of CNTs and SBS in modified asphalt materials, CNTs and SBS were used as modifiers to prepare CNTs/SBS composite-modified asphalt sealant. In order to evaluate the suitability of sealants prepared in this study for crack repair, the properties of sealants were tested. To address the insufficient research on fatigue performance of sealants in current research, the LAS test was carried out. Then evaluated the fatigue resistance performance of sealants, meanwhile, predicted the fatigue life of sealants. In addition, the rotational viscosity of sealants at different temperatures was tested, and the recommended construction temperatures of sealants were obtained according to the calculation results. The agitation heating method was used to simulate the construction aging of sealants. The softening point, cone penetration, elastic recovery, flow value, and cone penetration of sealants before and after aging were analyzed to investigate the use performance and age resistance of sealants.

## 2. Materials and Methods

### 2.1. Materials

An asphalt binder with the physical performance shown in Table 1 was selected as the base binder to prepare the sealants, and the information in Table 1 was provided by the manufacture (Sinopec Maoming Petrochemical Company, Guangdong, China). Star-shaped SBS polymers were obtained from Yanshan Petrochemical, China, and the 99% purity multi-walled CNTs were obtained from Shenzhen Sui Heng Technology Co., Ltd, Guangdong, China. The characteristics of SBS and multi-walled CNTs provided by the manufacturers were shown in Table 2 and Table 3. In addition, furfural extract oil (FEO) produced by Hebei Yisaiyuan Lubricant Co., Ltd., Xingtai, China, was selected as the compatibilizer in this study, because the organic aromatic compounds in FEO can improve the swelling effect of SBS and enhance the compatibility between SBS and asphalt [33].

### 2.2. Preparation of Samples

**Figure 1:**
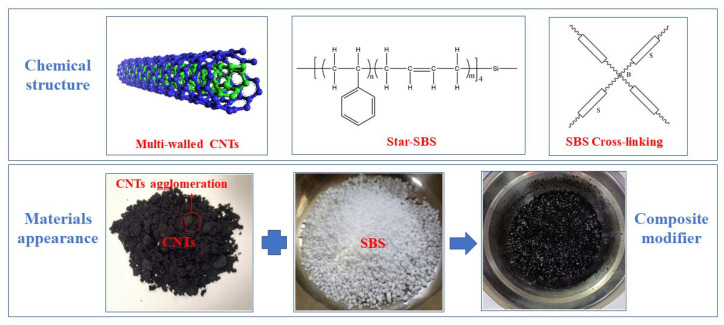
Schematic diagram of CNTs/SBS composite modifiers.

The microstructure and macro-morphology of SBS and CNTs are shown in Figure 1. As can be seen in the picture, CNTs are in the shape of black powders which are easy to form self-agglomeration. SBS are fluffy and porous white particles on the surfaces, and the SBS cross-linking represents the star structure of SBS. In order to avoid the agglomeration of CNTs affecting its dispersion effect in asphalt, and considering the high specific surface area of CNTs, it is easy to adsorb on the surface of fluffy porous materials. Therefore, in this study, SBS was added to the container containing CNTs at first and then stirred the two modifiers manually to obtain the CNTs/SBS composite modifier, as shown in Figure 1. Due to the different colors of the two modifiers, whether the two modifiers are mixed uniformly or not can be judged by the color changes before and after mixing the two modifiers. On the one hand, this method can fully adsorb CNTs on the surface of the SBS particles, on the other hand, the agglomerated CNTs can be dispersed by grinding between the SBS particles. Finally, the CNTs/SBS composite modifiers were added to the matrix asphalt. Then, the preparation of modified asphalt sealant is carried out. 

**Figure 2:**
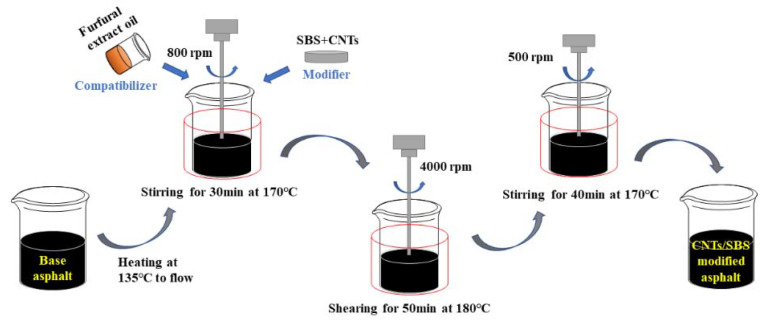
Schematic diagram of preparing CNTs/SBS-modified asphalt crack sealant.

Figure 2 shows a schematic diagram of the process of preparing CNTs/SBS-modified asphalt sealant using an electric mixer, high-speed shearing machine, and heating sleeve. The whole procedure of sample preparation is shown in Table 4. First, the base asphalt was heated to the flow state, and then FEO was added at 170 °C. After stirring evenly, CNTs/SBS composite modifier was added, and an electric mixer was used to stir and swell for 30 min at a speed of 800 rpm. Second, the temperature increased to 180 °C, and a high-speed shearing machine was used to shear for 50min at a speed of 4000 rpm. Finally, the temperature was reduced to 170 °C, and the mixture was stirred for 40 min using an electric agitator at a speed of 500 rpm. Then, CNTs/SBS-modified asphalt sealant was obtained. 

Too much CNTs will lead to the problem of agglomerations, so the contents of modifiers were worked out in combination with related research [18,33,34]. In this study, two kinds of modified asphalt sealants were prepared, in which the content of CNTs was 0.5 wt% and 1 wt%, respectively, the contents of SBS modifiers were 5 wt%, and FEO was 3 wt%. The above content is calculated as a percentage of the mass of matrix asphalt. The prepared samples were named CS-0.5 and CS-1 according to the content of CNTs.

### 2.3. Laboratory Tests

#### 2.3.1. Rotational Viscosity Test

The repairing effect of the sealant on the cracks is closely related to the construction temperature. Too low temperature will cause the sealant to be too viscous and difficult to fully penetrate into the cracks. Although high construction temperature can increase the fluidity of the materials, it may also accelerate the aging of the sealant and affect its road performance. The recommended construction temperature of sealant can be determined according to its viscosity at different temperatures. In addition, through the rotational viscosity test results at different temperatures, the high-temperature temperature sensitivity of the sealants can be analyzed. Therefore, the viscosity of two kinds of sealants was measured by using a Brookfield rotational viscometer (JTG: T 0625, Beijing, China) [35]. The test temperatures were 135 °C, 150 °C, 160 °C, 170 °C, 180 °C, 190 °C. The viscosity values of different rotational speeds at each temperature were measured by using No. 27 rotor, and the rotational speeds were 5 rad/s, 10 rad/s, 20 rad/s, and 50 rad/s, respectively.

#### 2.3.2. Conventional Properties

Different from the evaluation of matrix asphalt or modified asphalt, the evaluation index of sealants needs to carry out cone penetration test, resilience recovery test, and flow test. In this study, the softening point test at 5 °C (JTG: T 0606), the cone penetration test at 25 °C (JT/T: 6.2), the resilience recovery test at 25 °C (JT/T: 6.5), the flow test at 60 °C (JT/T: 6.4), and the penetration test at 25 °C (JTG: T 0604) were carried out. These tests were performed as laid down in the Ministry of Transport of the People’s Republic of China [35,36].

**Figure 3:**
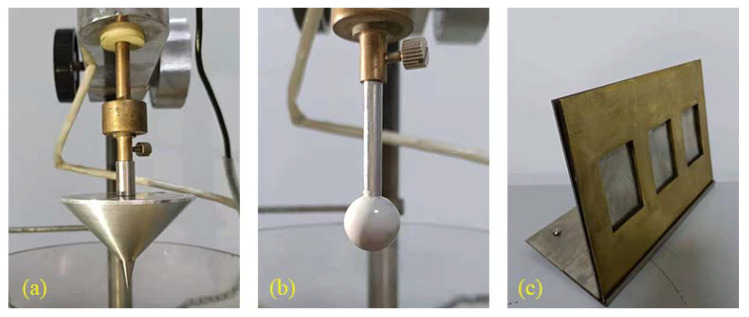
(**a**) Standard cone; (**b**) penetrating the ball; (**c**) mold and tripod.

**Figure 4:**
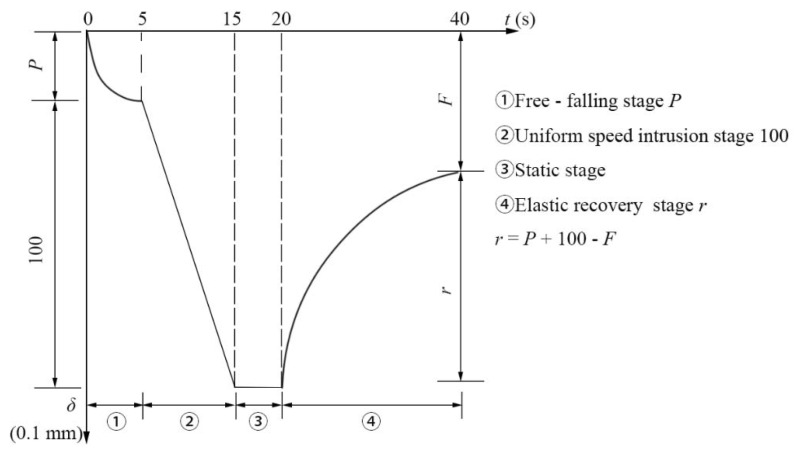
Schematic diagram of elastic recovery rate test process.

The cone penetration tests were carried out with a needle penetration meter, and the standard needle was replaced with a standard cone. As shown in Figure 3a, the test results reflect the softness and hardness of the material. The resilience recovery tests used a penetration meter (Nanjing Dongyong Shenfu Technology Co., Ltd., Nanjing, China), and replace the standard needle with a penetration ball, as shown in Figure 3b. The experiment was divided into four stages, and the schematic diagram of the test stages was drawn according to the “Hot-poured sealants for pavement” (JT/T 740-2015), as shown in Figure 4 [36]. The first stage is the free-falling stage, in which the penetration ball falls freely for 5 s, and the initial penetration amount P is recorded. The second stage is the uniform speed intrusion stage, in which the penetration ball is pressed into the sealant 10 mm at a constant speed within 10 s by pressing the connecting rod. Then, the total penetration volume is *P* + 100. The third stage is the static stage, where the penetration ball is fixed for 5 s. The last stage is the elastic recovery stage, in which the specimen is rebounded for 20 s and the residual penetration *F* is recorded. Then the resilience recovery rate of the sealant was calculated according to Equation (1). 

Both the flow test and the softening point test can reflect the high-temperature performance of the sealants. However, the flow test is to avoid the flow and sticky wheel problems of the sealant at high temperature, and the flow value of the sealants with high softening point is not necessarily up to the standard. First, the sample was poured into the brass mold frame as shown in Figure 3c, the dimensions of the mold frame are 60 mm × 40 mm × 3.2 mm (accurate to 0.1 mm). Then, place the treated sample together with the tripod in the oven at 60 °C for 5 h. The angle between the tripod and the horizontal direction is 75°. When the length of the specimen before and after the test is measured, the difference is the flow value.

(1) $$r=\frac{(P+100)-F}{(P+100)-P}\times 100=P+100-F,$$

where:

- r—Elastic recovery rate (%);
- P—Initial penetration (0.1 mm);
- F—Residual penetration (0.1 mm).

#### 2.3.3. Strain Sweep Test

**Figure 5:**
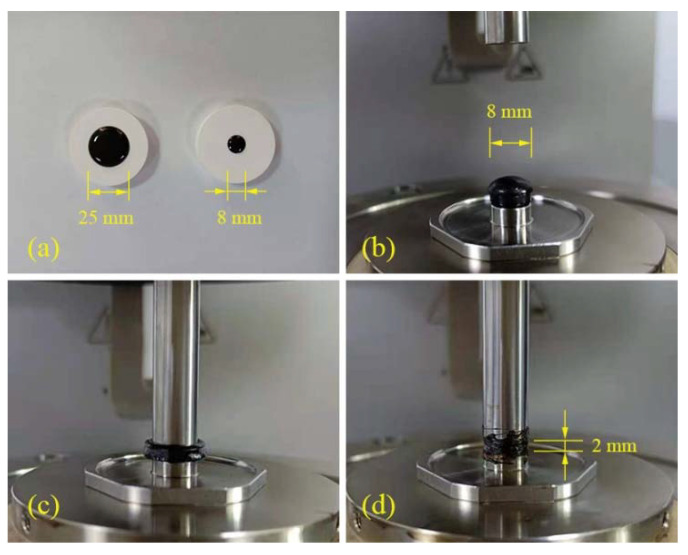
Preparation of DSR test samples (**a**–**d**).

Strain scanning test is used to determine the linear viscoelastic (LVE) region of modified asphalt sealant [37]. As shown in Figure 5a, the test specimens were poured into silicon molds. The testing instrument was Anton Paar MCR 102 Dynamic Shear Rheometer (DSR) using the 8 mm parallel plate geometry with a 2 mm gap setting, as shown in Figure 5b,c. In the experiment, the strain was controlled to change from 0.1% to 100%. The test temperature was 25 °C and the loading frequency was 10 Hz. Through the strain sweep test, the limit strain ($\gamma_{LVE}$) in the linear viscoelastic of the sealants at this temperature and frequency can be obtained.

#### 2.3.4. LAS Test

The standardized LAS procedure (AASHTO TP 101) was divided into two stages [32]. First, the frequency scanning test was carried out to test the non-destructive rheological properties of the samples. Then a linear amplitude scanning (LAS) test with a strain amplitude of 0.1% to 30% was carried out to test the damage tolerance of the sample. Therefore, in this study, the multi-temperature frequency sweep tests were carried out under a strain amplitude of 0.1% (≤$\gamma_{LVE}$), in the DSR using the 8-mm parallel plate geometry with a 2-mm gap setting. The test temperature condition was 20 °C, 25 °C, and 30 °C respectively. The loading frequency changes logarithmically and varies in the range of 0.1–100 rad/s [26,38]. Then, the strain-controlled loading mode was used to carry out the LAS test, the amplitude range increases linearly from 0.1% to 30%. The temperature condition of the test was 25 °C, the frequency was 10 Hz, and the scanning time was 300 s [31]. The test results recorded the average values of the peak shear strain, peak shear stress, phase angle, and dynamic shear modulus at each strain level. 

The test results of LAS were analyzed using the S-VECD model, with Wang et al. [32], Chen et al. [38] and other researchers’ study as a reference for S-VECD model and its application in asphalt materials. The key formulas of this method are as follows.

According to the principle of time-temperature superposition principle, the main curves of complex modulus were established by using the Christensen–Anderson–Marasteanu (CAM) model and Equation (2), and the shift factor $\alpha_{T}$ was calculated [34]. The slope m in double logarithmic coordinates was obtained by fitting according to Equation (3), and the parameter α was calculated by Equation (4).

(2) $$\left|G^{*}\right|=\frac{\left|G_{g}^{*}\right|}{{\left[1+f_{c}/{\left(\alpha_{T}\cdot f\right)}^{K}\right]}^{M/K}},$$

(3) $$log\left|G^{*}\right|(\omega )=mlog(\omega )+b,$$

(4) α=1+1/m,

where:

- $\alpha_{T}$—Shift factor;
- $\left|G^{*}\right|$—Complex modulus;
- $\left|G_{g}^{*}\right|$—Glass complex shear modulus;
- f—Actual loading frequency;
- $f_{c}$—Position fitting parameters of main curve related to loading frequency;
- M,K—Parameters of principal curve.

*S* is the state of internal damage, which is calculated according to Equation (5).

(5) $$S\left(t\right)={\sum_{i=1}^{N}\left[\frac{DMR}{2}{\left(\gamma_{p}^{R}\right)}^{2}\left(C_{i-1}-C_{i}\right)\right]}^{\frac{\alpha }{1+\alpha }}{\left(t_{Ri}-t_{Ri-1}\right)}^{\frac{1}{1+\alpha }},$$

where:

- $\gamma_{p}^{R}$—Peak pseudo strain, calculated according to Equation (6);
- DMR—Dynamic modulus ratio, calculated according to Equation (7);
- C—Pseudo stiffness, which is a function of the material with respect to the damage variable *S*, is calculated according to Equation (8);
- $t_{R}$—Reduced time, i is the number of cycles, calculated according to Equation (9).

(6) $$\gamma_{p}^{R}=\frac{\gamma_{p}\cdot {\left|G^{*}\right|}_{LVE}}{G_{R}},$$

(7) $$DMR={\left|G^{*}\right|}_{init}/{\left|G^{*}\right|}_{LVE},$$

(8) $$C\left(S\right)=\frac{\tau_{p}}{\gamma_{p}^{R}\cdot DMR},$$

(9) $$t_{Ri}=\frac{t_{i}}{\alpha_{T}},$$

where:

- $\gamma_{p}$—Peak shear strain;
- ${\left|G^{*}\right|}_{LVE}$—Complex modulus of linear viscoelastic stage;
- $G_{R}$—Reference shear modulus, with a value of 1;
- ${\left|G^{*}\right|}_{init}$—Initial modulus in amplitude scanning;
- $\tau_{p}$—Peak shear stress.

The relationship between the pseudo stiffness *C* and the damage variable *S* can be established according to the Equation (10), in which *C*_1_ and *C*_2_ are the fitting parameters.

(10) $$C\left(t\right)=1-C_{1}\cdot S{\left(t\right)}^{C_{2}},$$

The fatigue life $N_{f}$ of sealant is calculated and predicted according to Equation (11). Among them, $S_{f}$ is the damage parameter, which is calculated according to Equation (12), and *k* is the calculation parameter, which can be calculated according to Equation (13).

(11) $$N_{f}=\frac{f\cdot 2^{\alpha }\cdot S_{f}{}^{k}\cdot G_{R}^{2\alpha }}{k{\left(C_{1}C_{2}\right)}^{\alpha }{\left({\left|G^{*}\right|}_{LVE}\gamma_{p}\right)}^{2\alpha }},$$

(12) $$S_{f}={\left(\frac{1-C_{@PeakStress}}{C_{1}}\right)}^{\frac{1}{C_{2}}},$$

(13) $$k=1-\alpha C_{2}+\alpha .$$

## 3. Results

### 3.1. Rotational Viscosity

**Figure 6:**
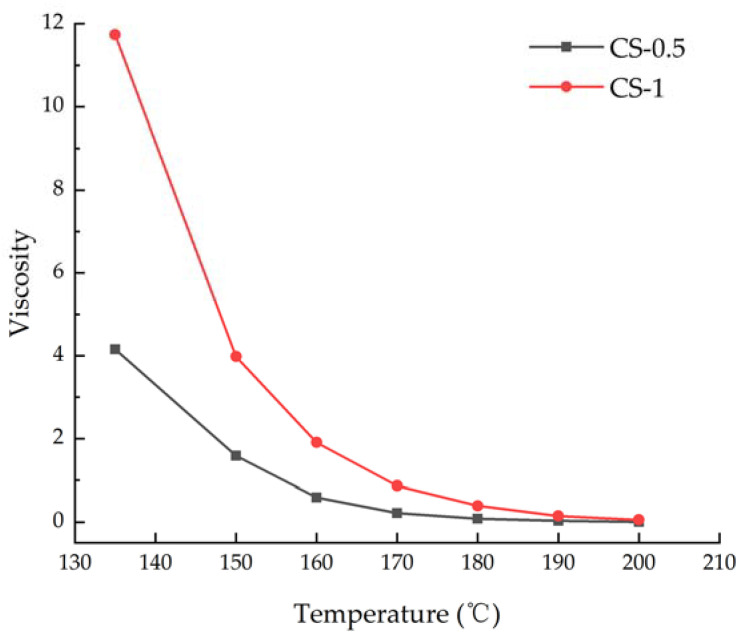
Viscosity–temperature curve diagram of grouting materials.

In this study, the viscosity and corresponding torque of different rotational speeds at the same temperature were recorded, and the results were processed logarithmically, and the regression equation was obtained by linear fitting. Finally, according to the regression equation, the corresponding viscosity value when the torque is 50% at the same temperature was calculated as the representative value of the viscosity of the measured temperature, as shown in Table 5 and Table 6. The viscosities of the two sealants at different temperatures were plotted into the viscosity–temperature curves shown in Figure 6. It can be seen from the figure that at the lower temperature of 135–160 °C, the content of CNTs has a significant effect on the viscosity of sealant, the viscosity of CS-1 is obviously higher than that of CS-0.5. This is due to the fact that the addition of CNTs increases the intermolecular interaction of asphalt materials, on the other hand, it is related to the surface energy of nano-materials [39,40]. Due to the strong van der Waals force on the surface of CNTs, it can prevent the destruction of the asphalt micelle structure during the experiment and increase the viscosity. In addition, the temperature sensitivity of the sealants is relatively high in the temperature range of 135–160 °C, and the viscosity of the sealant varies obviously with temperature [41]. At the temperature of 160–200 °C, the increase of CNTs content had no significant effect on the viscosity of the sealants. It shows the change of sealants’ viscosity was caused to the combined action of CNTs and the asphalt state.

For sealant CS-1, its viscosity exceeds 3 Pa·s at 150 °C, so it is difficult to ensure that the material can fully fill the cracks, which is not conducive to the construction effect. In addition, too high construction temperature will accelerate the aging of the sealant, resulting in damage to the road performance of the material. Therefore, considering the construction quality of the two sealants, the construction temperature was recommended to be 160–180 °C.

### 3.2. Conventional Properties

The sealant needs to be added to the sewing machine before construction, and the sealant should be sprayed into the crack after slotting and cleaning the crack. The construction efficiency of slotting and cleaning is lower than that of seam sealant, because the construction of seam sealant lags behind, and the heating time of sealant is prolonged. In this study, the mixer and the heating sleeve were used to simulate the construction aging of sealants. Then the performance indexes of sealant before and after aging were compared, and the influence of construction aging on sealant was studied. The aging temperature is determined according to the recommended construction temperature. In order to make the aging of the sealant more obvious, 180 °C was selected as the aging temperature in this study. According to the specification requirements of “JT/T 740—2015 Hot-poured sealants for pavement”, the technical specifications of ordinary sealants need to be met:

- Softening point: ≥80 (°C);
- Cone penetration: 50~90 (0.1 mm);
- Resilience recovery rate: 30~70 (%);
- Flow value: ≤5 (mm).

**Figure 7:**
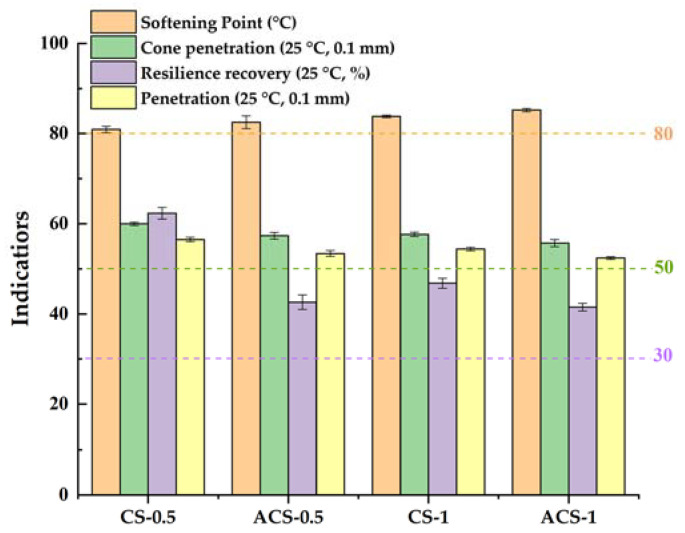
Conventional indicators of sealants.

As shown in Figure 7, both sealants meet the specification requirements before and after aging. Comparing the softening point of the two sealants, it can be found that the softening point of the sealants has been improved with the increase in the content of CNTs. In addition, the softening point of the two materials increased after aging. Through the cone penetration value, it can be found that the addition of CNTs caused the hardness of the sealants to improve, and the cone penetration value of CS-1 is slightly lower than that of CS-0.5. The changeable rule of penetration is similar to that of cone penetration.

After heating aging, the cone penetration and penetration of sealants were decreased due to the volatilization of light components in asphalt. The resilience recovery rate of CS-0.5 is significantly higher than that of CS-1, but the resilience recovery rate of CS-0.5 decreased more after aging. It shows that although the degradation of SBS after aging destructed the SBS network structure and reduces the resilience recovery rate of sealant, the addition of CNTs can prevent the degradation of SBS and inhibit the aging of materials to some extent [17,33]. The sealant with too-low resilience recovery rate is easy for embedding gravel, while the too-high resilience recovery rate is easy for forming secondary cracks. Comparing the resilience recovery of the two materials, the resilience recovery rate of CS-1 is close to the median technical index required by the specification and has better resistance to gravel and the ability to avoid the formation of secondary cracks.

The flow value is a technical index introduced to evaluate the high-temperature stability of the material. If the flow value of the material is not up to the standard, the sticky wheel phenomenon will easily occur at high temperatures. The flow values of the two materials before and after aging are shown in Table 7. The flow value of CS-1 is lower than CS-0.5. On the one hand, it is considered that the high-temperature performance of sealant has been improved with the increase of CNTs content, and it is not easy to flow. On the other hand, it is due to the higher CNTs content and higher viscosity of CS-1. The adsorption of CNTs on the surface of SBS increases the interfacial roughness of the polymer, thus improving the anchoring action of the interface between SBS and asphalt and reducing the flow value of the material at high temperatures [19].

### 3.3. Strain Sweep Test

**Figure 8:**
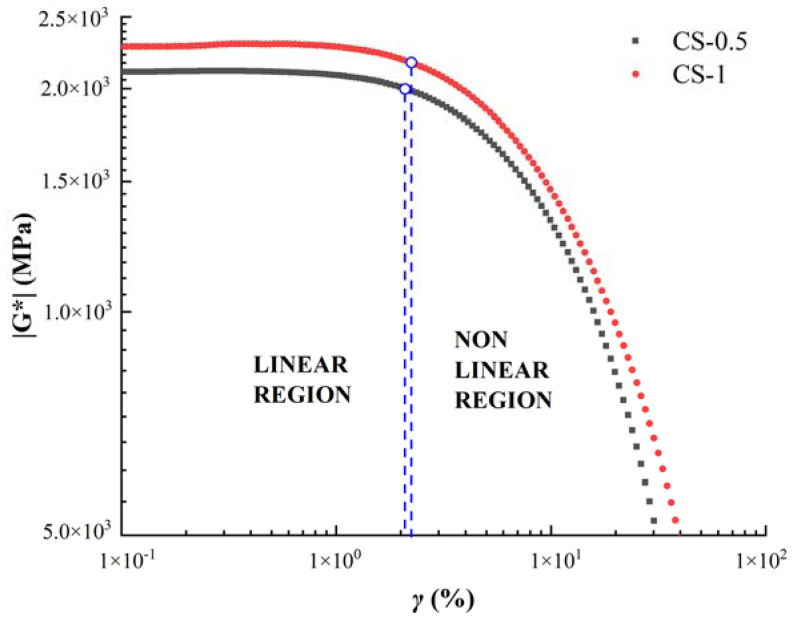
Strain sweep curves (CS-0.5 and CS-1 at 25 °C).

As a viscoelastic material, the rheological parameters such as complex modulus and phase angle of asphalt are defined under the condition of linear viscoelasticity [37]. Generally speaking, at the same temperature, the higher the frequency is, the smaller the linear viscoelastic range of asphalt is, at the same frequency, the lower the temperature is, the smaller the linear viscoelastic range of asphalt is. In order to obtain the LVE interval of asphalt under the most unfavorable conditions, the strain sweep test of this study was carried out under the condition of high frequency and low temperature. Therefore, in this study, 10 Hz is selected as the strain scanning test frequency, and the temperature condition is 25 °C. In order to verify the repeatability of the test, two tests were carried out for each sealant, and the test results were in good agreement. This paper only showed the results of a single test for observation. The strain-modulus curve is shown in Figure 8.

There is no definite value for the viscoelastic boundary of asphalt. According to the Strategic Highway Research Program (SHRP) study, the point corresponding to a decrease in the initial modulus equal to 5% was assumed as the LVE limit, which was marked in Figure 8 [14]. The viscoelastic range of the sealant was marked by the blue dashed line in Figure 8. The left side of the dotted line is the linear viscoelastic interval of the sealant, in which the stress–strain relationship of the material is only affected by temperature and frequency, while the right side is a nonlinear viscoelastic interval. It can be seen from Figure 8 that in the double logarithmic coordinate system, the complex shear modulus has a good linear relationship with the loading strain in the lower strain range, and the modulus change is not obvious. With the increase in strain, the linear relationship was destroyed and the complex shear modulus decreased greatly. Comparing the data curves of CS-1 and CS-0.5, it can be seen that although the complex modulus of sealant increased with the increase of CNTs content, the LVE range of sealant does not change significantly. The $\gamma_{LVE}$ of CS-1 is 2.26%, and the $\gamma_{LVE}$ of CS-1 is 2.16%.

### 3.4. LAS Test

#### 3.4.1. Master Curves

**Figure 9:**
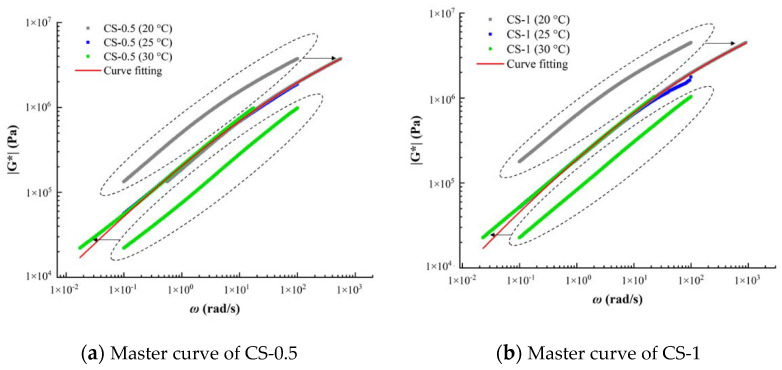
Master curves (**a**) CS-0.5 and (**b**) CS-1.

Through the frequency sweep test of LAS, the complex modulus curves of the two sealants at 20 °C, 25 °C, and 30 °C were obtained. Asphalt materials can show the same mechanical behavior under the action of equivalent temperature and equivalent time. Therefore, according to the time–temperature superposition principle, this paper takes the intermediate temperature 25 °C as the reference temperature and translates the complex modulus measured at other temperatures to the reference temperature to obtain the modulus master curve, as shown in Figure 9. The curves in the dotted line in the figure are the original data before moving. Using the CAM model, the complex modulus is fitted nonlinearly according to Equation (2) in Section 2.3.4, and the viscoelastic parameters and displacement factors of sealant are obtained. The results are shown in Table 8.

#### 3.4.2. Fatigue Performance Analysis

**Figure 10:**
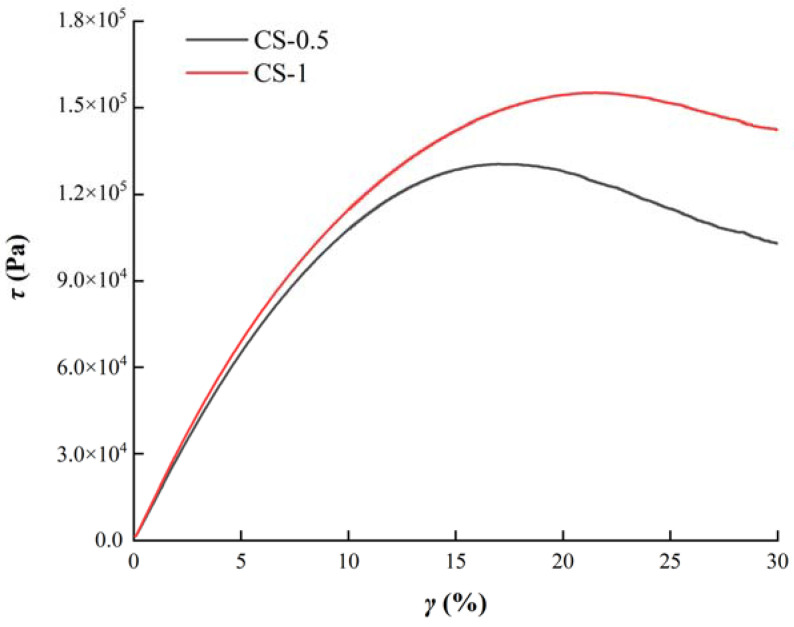
Stress–strain curves of sealants.

The stress–strain relationship curves of two sealants in the LAS test were shown in Figure 10. It can be seen that in the same range of strain amplitude, the stress value of CS-1 is higher than that of CS-0.5, and the peak strain of the CS-1 curve is also larger. In addition, with the increase of strain, the stress of CS-0.5 decreased more obviously.

**Figure 11:**
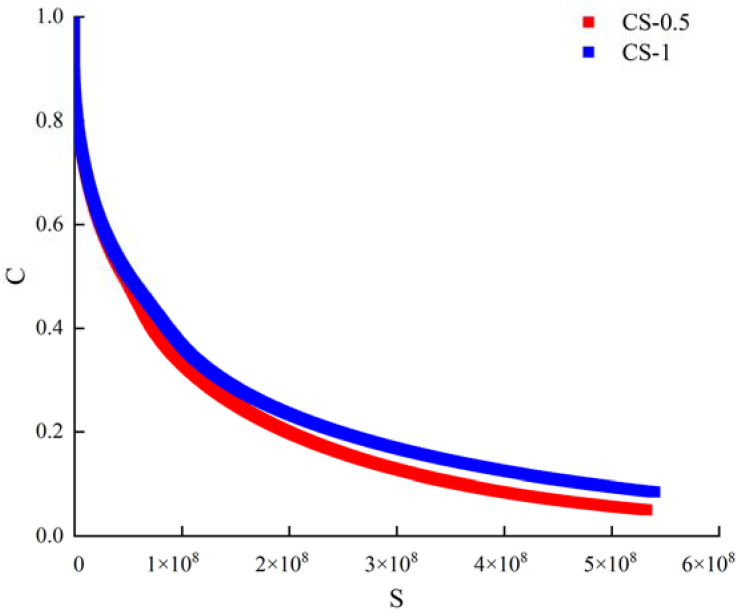
C-damage intensity curves based on LAS test.

The pseudo stiffness C represents the integrity of the material, and the damage variable *S* represents the damage degree of the material [26]. Figure 11 were C-damage strength curves drawn according to the material integrity coefficient C and the strength damage parameter S. It can be seen in the figure that the addition of CNTs can affect the damage evolution of the sealant. The C-damage strength curve of CS-1 is obviously higher than that of CS-0.5. It is considered that the material can maintain better integrity with the increase of CNTs content in the process of fatigue failure. In addition, the relationship between *C* and *S* can be expressed by the power-law Equation (10) in Section 2.3.4. In this section, the C-damage strength curves of the two sealants were non-linearly fitted, and the parameters *C*_1_ and *C*_2_ were obtained, as shown in Table 9. According to the correlation coefficient R^2^ of the fitting equation, we can see that the fitting result has a good correlation. At the same time, the fitting parameters *C*_1_ and *C*_2_ jointly determine the change rate of *C* with strength damage. For the same damage strength S, the smaller *C*_1_ and *C*_2_ are, the larger the corresponding pseudo stiffness *C* is, which means that the fatigue resistance of the sealant will be better. It shows that the addition of CNTs to the sealant can effectively improve the fatigue resistance of the material.

#### 3.4.3. Predicted Fatigue Life from LAS Test through S-VECD Approach

**Figure 12:**
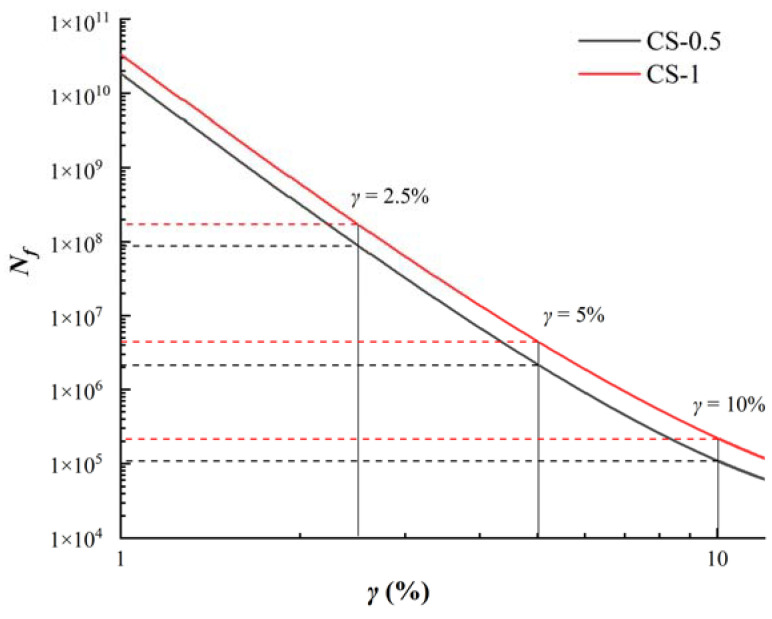
The fatigue life of sealants under different strains.

In order to reflect the fatigue properties of the two sealants directly, the fatigue life *N_f_* of the two sealants under different strain conditions were calculated according to Equation (11) in Section 2, as shown in Figure 12. The corresponding damage strength under peak stress is calculated. In the figure, the fatigue lives of each material were marked under 2.5%, 5%, 10% strain conditions. It can be observed that CS-1 has a larger *N_f_* value, which is 1.7 times that of CS-0.5 *N_f_.* It showed that in the asphalt sealant composite incorporating CNTs/SBS, compared with the sealant containing 0.5% CNTs, the sealant containing 1% CNTs has a better fatigue resistance performance. The fatigue damage process of sealant can be divided into two stages: first, micro-crack damage is formed under low strain loading, and then, with the increase of strain, micro-crack extends to macro-crack. Therefore, the positive effects of CNTs on the fatigue resistance of sealants can be explained from two aspects. When loaded with low strain, CNTs enhanced the interface compatibility between the polymer and asphalt and can absorb and disperse external loads [42]. With the increase of strain, due to the special physical structure of CNTs, the interface pull-out behavior and crack bridging are produced in the crack propagation stage, which prevents the crack propagation to a certain extent [15].

## 4. Conclusions

In this paper, CNTs/SBS composite-modified asphalt sealant was prepared by the high-speed shearing method, and the CNTs content in the sealant was 0.5 wt% and 1 wt%, respectively. The performance of the sealant was studied using rotational viscosity, softening point, penetration, elastic recovery, flow value, and LAS, including the high-temperature performance, anti-aging performance, and fatigue resistance of the sealant. The research conclusions are as follows:

- The sealant with high CNTs content has higher viscosity. There is an obvious difference in viscosity between CS-1 and CS-0.5 in the temperature range of 135 °C~160 °C, and the viscosity difference decreases with the increase of temperature. At the same time, the viscosity value also depends on the flow state of asphalt itself, with the increase of temperature, the effect of CNTs modifier on the viscosity of sealant is no longer significant.
- The softening point, taper penetration, elastic recovery, and flow value of the sealant all meet the requirements of the specification. CNTs has a positive effect on the high temperature properties of sealants. The flow value of CS-1 is 0.9 mm, and the flow value of CS-0.5 is 1.9 mm.
- The sealant with high CNTs content has better age resistance performance. After simulating construction aging, the decrease of resilience recovery rate of CS-1 is lower than that of CS-0.5. CNTs can prevent the thermal aging of sealant to a certain extent, reduce the hardening degree of sealant, and maintain the elasticity of sealant.
- In the linear viscoelastic range of sealants, there is a good linear relationship between logarithmic loading strain and logarithmic complex shear modulus of sealants. CNTs increased the complex modulus of sealant, but there are no obvious effects on the linear viscoelastic range of sealant.
- The calculation of N*_f_* value of fatigue life provides a method to quantitatively evaluate the fatigue resistance of sealants. The fatigue life of sealants under different strain conditions can be predicted by calculation, and the fatigue life of CS-1 is higher than that of CS-0.5.

It should be mentioned that in the calculation presented in this paper, the failure criterion is defined as the damage strength of the maximum shear stress. In the study of fatigue resistance performance of sealants, the definition of failure criterion affects the parameters of fatigue model. Some researchers have proposed to take a certain reduction of stiffness as the failure standard. Therefore, the selection of failure criteria needs to be further studied, so that it can be applied to a wider range of experimental and research conditions. This study used parallel-group tests to verify the repeatability of the test results. There is a limitation for obtaining general conclusions due to the limited amounts of tests in this paper, but it revealed a correlation between sealant performances and factors affecting performances change, and provided research foundation and reference basis. In future research, it is necessary to considering adding contrast groups and parallel groups to make the test results more repeatable and universal. In addition, the macroscopic properties of sealants were studied in this paper. At present, most studies are focused on the macro-properties of CNTs-modified asphalt, but there are a few studies on the modification mechanism of nanomaterials. It is also necessary to further study the microscopic behavior of CNTs in asphalt and have a systematic understanding of the modification mechanism of CNTs.

## Figures and Tables

**Table 1:** Basic performances of neat asphalt.

Item	Unit	Standard Values	Values
Penetration (25 °C, 100 g, 5 s,)	0.1 mm	80–100	84
Softening point	°C	≥45	46.0
Dynamic viscosity (60 °C)	Pa·s	≥160	178
Ductility (10 °C)	cm	≥20	>100
Ductility (15 °C)	cm	≥100	>100
RTFOT residue			
Quality change	%	−0.8–0.8	−0.112
Residual penetration ratio	%	≥57	62.4
Residual ductility (10 °C)	cm	≥8	11.9

**Table 2:** Main characteristics of SBS.

Styrene (%)	Butadiene (%)	Tensile Strength (MPa)	Molecular Weight (g/mol)
40	60	26	160,000

**Table 3:** Main characteristics of CNTs.

Inside Diameter (nm)	Outer Diameter (nm)	Average Length (μm)	Surface Area (m^2^/g)	Density (g/cm^3^)	Carbon Purity (%)	Exterior
3–5	8–15	8–14	≥250	0.01	>99	Black powder

**Table 4:** Summary of sample preparation procedures.

Procedures	Temperature (°C)	Speed (rpm)	Time (min)
Stirring	170	800	30
Shearing	180	4000	50
Stirring	170	500	40

**Table 5:** Viscosity fitting results of CS-0.5.

Temperature (°C)	Regression Equation	R^2^	Viscosity (Pa∙s)
135	y = −0.0034x + 3.7886	0.967	4.155
150	y = −0.0072x + 3.5617	0.896	1.591
160	y = −0.0148x + 3.5104	0.917	0.589
170	y = −0.0217x + 3.4231	0.929	0.218
180	y = −0.0284x + 3.3381	0.931	0.083
190	y = −0.0343x + 3.2589	0.930	0.035
200	y = −0.0494x + 3.2340	0.938	0.006

**Table 6:** Viscosity fitting results of CS-1.

Temperature (°C)	Regression Equation	R^2^	Viscosity (Pa∙s)
135	y = −0.0033x + 4.2345	0.895	11.735
150	y = −0.0071x + 3.9550	0.912	3.981
160	y = −0.0114x + 3.8507	0.910	1.909
170	y = −0.0165x + 3.7639	0.864	0.869
180	y = −0.0224x + 3.7119	0.927	0.391
190	y = −0.0308x + 3.7119	0.937	0.149
200	y = −0.0378x + 3.6307	0.938	0.055

**Table 7:** Flow value of sealants.

Samples	CS-0.5	ACS-0.5	CS-1	ACS-1
Flow value (mm)	1.9	1.7	0.9	0.4

**Table 8:** Master curves fitting parameters.

Samples	Fitting Parameters						
	|G^*^_g_|	*f_c_*	$\alpha_{T}$	*K*	*M*	*R* ^2^	*m*
CS-0.5	30649830.18	3.77	1.49	0.22	0.73	0.99	0.510
CS-1	34639718.46	3.09	1.32	0.21	0.79	0.99	0.511

**Table 9:** Fitting parameters.

Samples	Fitting Parameters			
	*C*_1_	*C*_2_	*R* ^2^	*k*
CS-0.5	3.79E-3	0.278	0.99	3.13
CS-1	3.77E-3	0.276	0.99	3.14

## Data Availability

The data presented in this study are available on request from the corresponding author. The data are not publicly available due to the project restrictions.
